# Editors at the frontier: exploring advanced optical engineering in the UK

**DOI:** 10.1038/s41377-026-02292-8

**Published:** 2026-04-20

**Authors:** Siqiu Guo, Xiangqian Jiang

**Affiliations:** 1 Light Publishing Group, Changchun Institute of Optics, Fine Mechanics and Physics, Chinese Academy of Sciences, 3888 Dong Nan Hu Road, Changchun, 130033 China; 2 Ji Hua Laboratory, 28 Huandao South Road, Foshan, 528200 China; 3 EPSRC Future Metrology Hub, Centre for Precision Technologies, University of Huddersfield, Huddersfield, HD1 3DH UK

**Keywords:** Optics and photonics, Physics

## Abstract

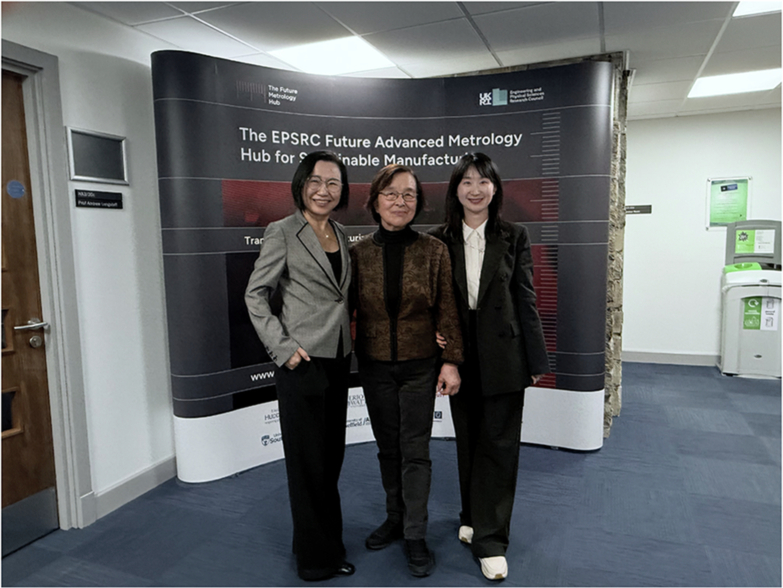

At the invitation of Professor Dame Xiangqian (Jane) Jiang DBE FREng—Professor of Precision Metrology at the University of Huddersfield, Director of the EPSRC Future Metrology Hub, and a globally recognized leader in precision engineering—Prof. Yuhong Bai, the founder of Light Publishing Group, the founder and Executive Editor-in-Chief of *Light: Science & Applications (Light)*, and Dr. Siqiu Guo, Deputy Editor-in-Chief of the Light Publishing Group, visited the University of Huddersfield in early March 2026.

During the visit, Prof. Jiang introduced the research landscape of the Centre for Precision Technologies at Huddersfield, which underpins the EPSRC Future Metrology Hub—a world-leading initiative in advanced metrology, precision manufacturing, and surface engineering. The laboratory environment vividly reflected the convergence of fundamental science and industrial application, with ultra-precision measurement systems, optical interferometry, and advanced sensing technologies working in concert to push the boundaries of accuracy and reliability. These efforts are not isolated technical pursuits; rather, they are increasingly embedded in manufacturing processes, enabling real-time measurement and control, where light serves as both a tool for precision metrology and a carrier of information. This vision resonates closely with the philosophy underpinning the *Light* brand, as articulated by Prof. Bai, where the integration of fundamental science, technological innovation, and real-world application has been a central guiding principle. Subsequently, Prof. Bai introduced the positioning and future development of the Light Publishing Group, expressing a strong expectation for further international collaboration.

More importantly, this visit provided an opportunity to engage directly with researchers at the moment when knowledge is still in formation—before it is distilled into manuscripts. Conversations with faculty members, postdoctoral researchers, and students revealed not only the technical challenges they are addressing, but also the conceptual shifts underway in the field: the transition from single-device optimization to system-level integration, from deterministic metrology to data-driven intelligence, and from laboratory prototypes to industrial-scale deployment. These insights, often less visible in published literature, are essential for understanding where the field is heading.

This experience reinforces a broader transformation in the role of scientific editors. Traditionally positioned as evaluators of completed work, editors must now evolve into active participants in the scientific ecosystem. Visiting laboratories such as those at Huddersfield enables editors to observe how innovation unfolds in practice —how hypotheses are tested, how unexpected results reshape research directions, and how interdisciplinary collaboration gives rise to new ideas. In this sense, the editorial desk extends into the laboratory, and the boundary between publishing and research becomes increasingly close and interactive.

For *Light*, such engagement serves multiple strategic purposes. First, it allows us to maintain a first-hand awareness of emerging directions in optical science and engineering, particularly in areas where precision technologies intersect with photonics. Second, it strengthens intellectual trust between the journal and leading research groups, creating a foundation for attracting transformative contributions. Third, it opens avenues for proactive editorial initiatives, including invited articles, thematic collections, and collaborative academic activities that can capture innovation at its early stages.

At a broader level, this visit reflects an evolving understanding of internationalization for Chinese scientific journals. True global engagement is not merely a matter of language or editorial board composition; it requires sustained, in-depth interaction with the world’s leading research environments. By entering laboratories, participating in scientific dialog, and understanding diverse research cultures, journals can position themselves as active nodes in the global circulation of knowledge. In doing so, they contribute not only to dissemination but also to the understanding and shaping of scientific trajectories (Figs. 1–3).

**Fig. 1:**
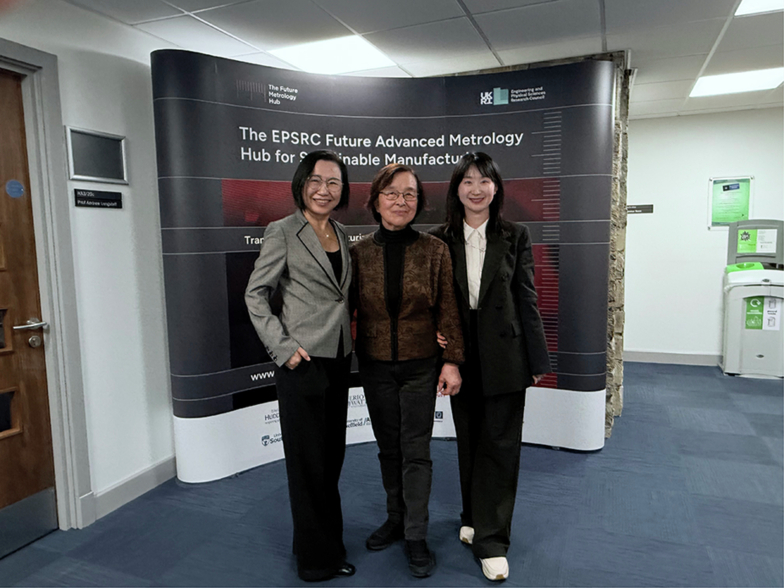
Prof. Yuhong Bai and Dr. Siqiu Guo with Prof. Xiangqian Jiang

**Fig. 2:**
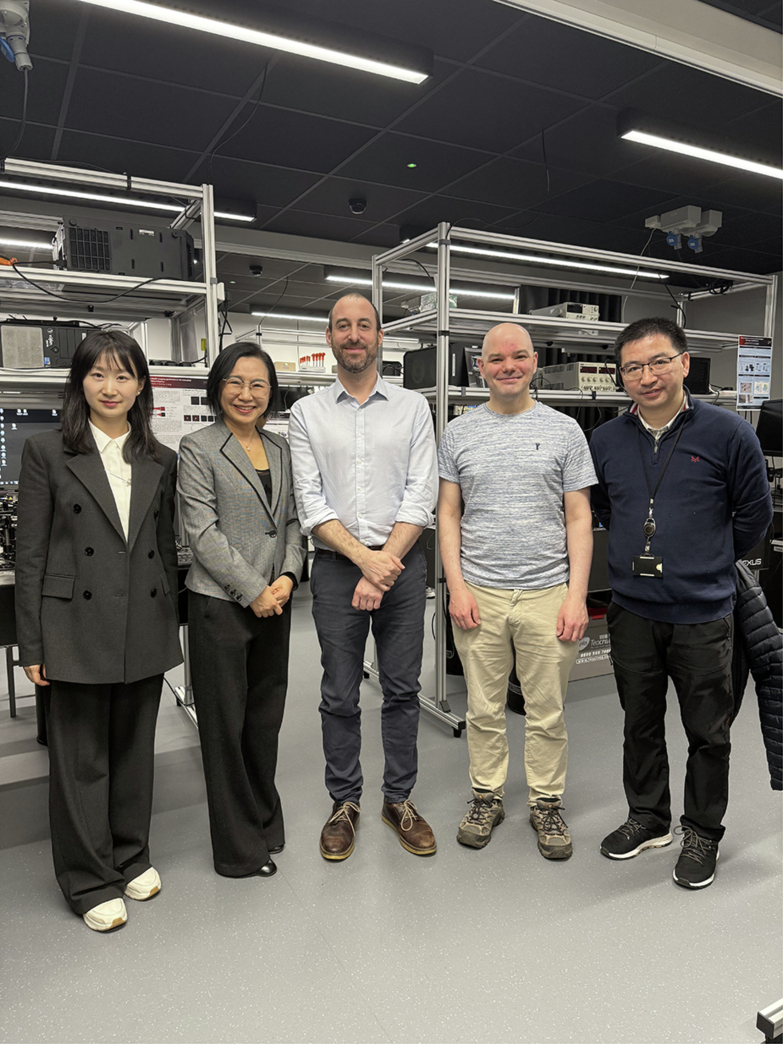
Visit to Prof. Xiangqian Jiang’s lab (from left to right: Dr. Siqiu Guo, Prof. Yuhong Bai, Dr. Haydn Martin, Dr. Andrew Henning, and Dr. Wenhan Zeng)

**Fig. 3:**
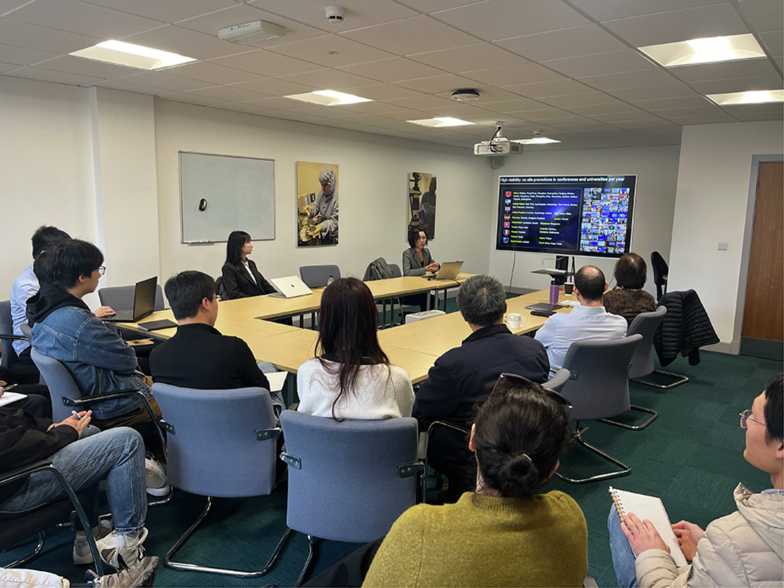
Prof. Yuhong Bai in discussion with faculty members and students from the Centre for Precision Technologies

The discussions at Huddersfield further highlighted a key trend in contemporary optical science: the increasing importance of precision. As photonics technologies move toward real-world applications—whether in advanced manufacturing, quantum technologies, or intelligent sensing—the demand for accuracy, stability, and traceability becomes paramount. Precision engineering and optical science are no longer parallel disciplines; they are deeply intertwined. Capturing this convergence requires editorial vision that recognizes emerging intersections and supports interdisciplinary narratives.

As *Light* continues to evolve, visits such as this will play a critical role in informing our editorial strategy. By staying close to the environments where knowledge is created, we aim to better identify frontier topics, engage leading researchers, and support the global photonics community. Ultimately, the mission of a journal is not only to record scientific progress but to illuminate the pathways through which that progress emerges.

